# The X-Rays and Artificial Teeth

**Published:** 1900-03-15

**Authors:** Henry Blandy


### THE X-RAYS AND ARTIFICIAL TEETH.

In the *Record* of December, 1899, is an extract from the *British Medical Journal*, recounting the death of a woman in Paris from swallowing a portion of her artificial teeth. It states that the X rays were of no use in locating the position of the plate, on which were two teeth. The editor of the *Record* appends the note: "If the plate were made of vulcanite it would be transparent to the X rays, and hence could not be localized." I have the honor and pleasure of doing the X-ray experiments at the General Hospital, Nottingham, and to test this statement was, of course, extremely easy. I feel sure the editor will forgive me for doubting the accuracy of his note, and in the interest of science publish these little experiments. Upon a Landall's Roentgen 10-inch by 8-inch plate I arranged eleven old vulcanite plates, one metal one, one bit of India rubber tube, and one elastic band, which encloses my numbering device (made of card and leather) for negatives—this is 223. The red and pink vulcanite plates show as distinctly as the metal one. The black vulcanite less so. But in all cases the teeth are perfectly defined, with their platinum pins. The tube, and even the elastic ring surrounding the leather case, are also plainly visible. I then bandaged one of the plates on the throat of a boy—taking the negative right through the neck, from front to back. The plate of vulcanite appears there distinctly also. The thickness of neck or part is merely a question of length of exposure and development. I write this at once, in order that the X-rays may not be discredited by the failure of the Parisian operator, or by the foot note of our esteemed editor; or, perhaps, the next person who happens to swallow artificial teeth might be given over to cuts in the dark and exploratory incisions, from which X-rays now save many patients, in cases of foreign bodies.—*Henry Blandy, L.D.S., Dental Record.*

